# Hybrid modeling approach for mode-locked laser diodes with cavity dispersion and nonlinearity

**DOI:** 10.1038/s41598-021-89508-6

**Published:** 2021-05-11

**Authors:** Stijn Cuyvers, Stijn Poelman, Kasper Van Gasse, Bart Kuyken

**Affiliations:** 1 Photonics Research Group, INTEC Department, Ghent University - imec, 9052 Ghent, Belgium; 2 Center for Nano- and Biophotonics, Ghent University, Ghent, Belgium

**Keywords:** Engineering, Optics and photonics

## Abstract

Semiconductor-based mode-locked lasers, integrated sources enabling the generation of coherent ultra-short optical pulses, are important for a wide range of applications, including datacom, optical ranging and spectroscopy. As their performance remains largely unpredictable due to the lack of commercial design tools and the poorly understood mode-locking dynamics, significant research has focused on their modeling. In recent years, traveling-wave models have been favored because they can efficiently incorporate the rich semiconductor physics of the laser. However, thus far such models struggle to include nonlinear and dispersive effects of an extended passive laser cavity, which can play an important role for the temporal and spectral pulse evolution and stability. To overcome these challenges, we developed a hybrid modeling strategy by unifying the traveling-wave modeling technique for the semiconductor laser sections with a split-step Fourier method for the extended passive laser cavity. This paper presents the hybrid modeling concept and exemplifies for the first time the significance of the third order nonlinearity and dispersion of the extended cavity for a 2.6 GHz III–V-on-Silicon mode-locked laser. This modeling approach allows to include a wide range of physical phenomena with low computational complexity, enabling the exploration of novel operating regimes such as chip-scale soliton mode-locking.

## Introduction

Semiconductor-based Mode-Locked Lasers (MLLs), emitting coherent ultrashort^1–4^ optical pulses, are an important class of chip-scale comb generators with numerous applications in fundamental science and technology^5–8^. While a number of such devices have already been demonstrated^1–3,9–15^, their design remains challenging as it is largely based on simple rules of thumb and hence lacks predictability^4^. It is evident that advanced modeling techniques are therefore indispensable to facilitate the development process and advance the understanding of the complex mode-locking dynamics^16–20^. Ideally, such a model can provide a set of design rules to acquire some targeted device parameters such as pulse duration, output power and comb shape. Furthermore, it is desirable that the MLL model not only incorporates the necessary physical details, but also minimizes the computational workload in order to serve as a design aid and enable parametric studies.

In the past decades, a wide variety of modeling techniques for MLLs have been presented, which, as proposed in^21^, can be categorized in two distinct classes: distributed models and discrete models. Distributed models average the effects on the circulating pulses so that a single partial differential equation can be employed to describe the MLL. It is often based on the Haus’s master equation^22^, the cubic quintic Ginzburg–Landau equation^23^ or the Swift–Hohenberg equation^16,24^. Although these models allow for analytical solutions and enable the study of pulse dynamics, they assume that the pulse is near equilibrium and only undergoes mild changes when traveling inside the laser cavity^25,26^. MLLs with strong gain and high losses in each roundtrip can therefore not be appropriately represented. Moreover, these equations employ generic formalisms to describe gain and absorption and are hence unable to grasp complex semiconductor physics that can greatly affect the gain and saturable absorber characteristics^20,21^. In contrast, discrete models can be seen as an approach where each component of the laser cavity is modeled separately. This does not necessarily mean a different set of equations is utilized for the various laser components, rather it often implies the parameters in the equations are distinct for different components. In other words, the gain, saturable absorption, etc. happen in different sections of the device and the assumption that the pulse is near equilibrium is therefore eliminated^21^. Such discrete models are typically based on delay differential equations^25–27^, a finite difference time domain description of the electromagnetic field^28^, the full Maxwell–Bloch equations^16,29–31^, or traveling-wave models^19,20,32–34^.

In recent years, Traveling-Wave Models (TWMs) have mostly been favored for semiconductor-based MLL modeling because they can incorporate the rich physics of semiconductors while limiting the computational workload under to the slowly-varying envelope approximation^17,19,32^. However, existing TWMs are not geared to incorporate nonlinear and dispersive effects of a long extended passive laser cavity^18,19,32^. Although recent work successfully included Kerr-nonlinearity and chromatic dispersion in a TWM^20^, these computationally intensive models target single-section diode mode-locked lasers and are consequently not suitable to aid the design of extended cavity semiconductor MLLs or to perform parametric studies. This is particularly troublesome when simulating low-repetition-rate MLLs, which have a long extended passive cavity as compared to the length of the active semiconductor section. For these devices, dispersive and nonlinear effects of the passive waveguides can become important. Moreover, low-repetition-rate integrated mode-locked lasers are becoming increasingly relevant in the pursuit of lower repetition rates and low noise performance^4,9,10^. Yet the ability to efficiently model such devices while including relevant nonlinear and dispersive effects of the long extended passive cavity has not been demonstrated by existing traveling-wave models. On the other hand, the well-established split-step Fourier method is well-suited to rapidly simulate pulse evolution in passive waveguides with arbitrary dispersion and nonlinearity^35–37^. It could therefore be advantageous to combine a TWM for the semiconductor laser sections with a split-step Fourier approach for the extended passive laser cavity.

In this work, we demonstrate a first implementation of such a hybrid simulation strategy and include nonlinear and dispersive effects of the extended passive laser cavity with low computational penalty. Furthermore, the method is used to simulate a 2.6 GHz III–V-on-Silicon Mode-Locked Laser. In particular the impact of the Kerr nonlinearity, dispersion, two-photon and free-carrier absorption of the extended passive laser cavity on the laser performance is discussed.

## Results

### Traveling-wave model equations

The TWM proposed here is applied to model a previously demonstrated III–V-on-Silicon anti-colliding MLL with a 2.6 GHz repetition rate^10,38^. The laser consists of a 14  mm long silicon waveguide cavity, a ${850}\,\upmu$m Semiconductor Optical Amplifier (SOA) gain section and a ${60}\,\upmu$m Saturable Absorber (SA) separated with an unbiased ${30}\,\upmu$m ISOlation section (ISO) from the gain region. Although several elaborate TWMs have been proposed in literature for semiconductor quantum well and quantum dot lasers, such as^20^ and the open source model Freetwm^19^, a simpler TWM is adopted here to demonstrate the hybrid modeling concept. It is nevertheless straight forward to extend the presented approach to any other TWM in order to include more physical details. Assuming the MLL pulse width is significantly larger than the intraband relaxation time of the semiconductor medium, one can describe the temporal and spatial evolution of two counterpropagating waves with amplitudes $A^{\pm }$ (units of $\sqrt{{\text {W/m}}^{2}}$) in the semiconductor laser sections as^32,39^

1 $$\begin{aligned} \frac{\partial A^{\pm }}{\partial t}\pm \frac{\partial A^{\pm }}{\partial z}=\frac{j \omega _{0}v_{g}\Gamma }{2n_{eff}c}\chi A^{\pm } - \frac{\beta }{2}A^{\pm } \end{aligned}$$

where $v_{g}$ represents the group velocity of light, $\Gamma$ the Multiple Quantum Well (MQW) optical confinement factor, $n_{eff}$ the effective modal index, c the speed of light, $\omega _{0}$ the angular frequency of operation and $\chi$ the electrical susceptibility. Furthermore, the spatial variable z was divided by the group velocity to yield a dimension of time. The term containing $\beta$ models the internal losses in the semiconductor material. Furthermore, the carrier density N can be approximated as^32,39^

2 $$\begin{aligned} \frac{\partial N}{\partial t} = \frac{I}{qV} - \frac{N}{\tau }+\frac{\Gamma {\text {Im}}(\chi (N))}{\hbar n_{eff}c} |A|^{2} \end{aligned}$$

where I is the injected current, q is the electron charge, V is the active volume and $\tau$ represents the carrier lifetime. Assuming a parabolic band structure, low temperature, charge neutrality within the quantum well, and k-vector independent intraband relaxation rates, one can express the frequency-dependent electrical susceptibility as^17,18,40^

3 $$\begin{aligned} \chi (\omega ,N)=-\chi _{0} \left[ 2ln\left( 1-\frac{\gamma \frac{N}{N_{t}}}{\omega -\Omega _{g}+j\gamma }\right) -ln\left( 1-\frac{\Omega _{T}}{\omega -\Omega _{g}+j\gamma }\right) \right] , \end{aligned}$$

where $N_{t}$ is the transparency carrier density, $\Omega _{g}$ is the bandgap offset, $\gamma$ is the intraband relaxation rate, $\Omega _{T}$ is the angular frequency of the top of the energy band and $\chi _{0}$ is a material gain parameter. To eliminate the frequency-dependency of the susceptibility, one can approximate the susceptibility as a frequency-independent quantity $\chi (\omega =\omega _{0},N(z,t))$ by adding a separate spectral filter to the model. This approximation can be motivated by noting that Bragg gratings are utilized as cavity mirrors in the MLL under consideration, causing strong spectral shaping. Here, a Lorentzian filter is incorporated at the output facet of the laser, in correspondence to what was proposed in previous work^32^. In case a more accurate model for gain dispersion is desired, the lumped filter could be replaced by a distributed implementation, similar to what has been demonstrated in^41,42^. This is particularly important for laser topologies without narrowband reflectors where the gain dispersion of the semiconductor medium plays a salient role^41^.

**Figure 1:**
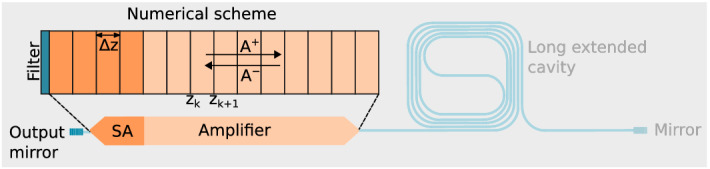
Traveling-wave model numerical scheme for the mode-locked laser’s active semiconductor sections. The sections are discretized in segments of optical length $\Delta z=\Delta t$. A Lorentzian filter is used at the output facet to model the gain bandwidth and spectral shaping of the gratings. *SA* saturable absorber, $A^{\pm }$: amplitudes of the counterpropagating waves in the cavity.

In order to solve the aforementioned set of equations, a numerical scheme is used where the semiconductor section is discretized into K segments of normalized length $\Delta z=\Delta t$, where $\Delta t$ is the time required for light to travel over one segment and $\Delta z$ is the physical length of the segment normalized with the group velocity of light. A schematic of the TWM numerical scheme is depicted in Fig. 1. Combining Eqs. (1) and (3), the wave solutions in each segment $z_{k}\le z \le z_{k+1}$ are given by^32^

4 $$\begin{aligned} A^{+}(t+\Delta t,z_{k+1})\approx & {} A^{+}(t,z_{k})exp\left[ -\frac{\beta \Delta z}{2}+\frac{j\omega _{0}\Gamma v_{g}}{2 n_{eff}c} \cdot \int _{z_{k}}^{z_{k+1}} \chi (N(z,t))dz\right] \end{aligned}$$

5 $$\begin{aligned} A^{-}(t+\Delta t,z_{k})\approx & {} A^{-}(t,z_{k+1})exp\left[ -\frac{\beta \Delta z}{2}+\frac{j\omega _{0}\Gamma v_{g}}{2 n_{eff}c} \cdot \int _{z_{k}}^{z_{k+1}} \chi (N(z,t))dz\right] \end{aligned}$$

The integrals can be evaluated numerically by using a simple trapezoidal approximation. Furthermore, an expression for the carrier density can be formulated based on Eq. (2) and a first order Euler scheme

6 $$\begin{aligned} N(z_{k},t) \approx N(z_{k},t-\Delta t)+\Delta t \cdot \left[ \frac{I}{qV} - \frac{N(z_{k},t-\Delta t)}{\tau }+\frac{\Gamma }{\hbar n_{eff}c} \mathfrak {I}\{\chi (N(z_{k},t-\Delta t))\} \cdot |A^{+}(t,z_{k})+A^{-}(t,z_{k})|^{2}\right] \end{aligned}$$

The stimulated recombination term was chosen to scale with the local field intensity $|A^{+}+A^-|^{2}$ rather than the photon density $|A^+|^{2}+|A^-|^{2}$, as was experimentally observed from gain-coupled DFB lasers^43,44^. Furthermore, for this first demonstration of a hybrid modeling approach, a large TWM step size is chosen compared to the optical wavelength, leading to an averaging effect. Coupled-wave equations, carrier diffusion and carrier gratings are therefore not included here and will be addressed in future work. Finally, a boundary condition can be defined for the left laser facet by convolving the backward propagating wave with the Lorentzian spectral filter f(t)^32^

7 $$\begin{aligned} A^{+}(t+\Delta t,0)=\sqrt{r_{1}} \Delta t \sum _{m=0}^{M}f(m)A^{-}(t-(m-1)\Delta t,0) \end{aligned}$$

where $r_{1}$ describes the left facet reflectivity and M is here taken as $\left\lceil {\frac{10\,ps}{\Delta t}}\right\rceil$. Alternatively, the convolution can be implemented using a first-order IIR filter of the form $A^{+}(t+\Delta t,0)=\sqrt{r_{1}} \left[ a \cdot A^{+}(t,0) +b \cdot A^{-}(t+\Delta t,0) \right]$. The right boundary condition (corresponding with the right mirror of Fig. 1) is simply defined as $A^{-}(t+\Delta t,L)=\sqrt{r_{2}} \cdot A^{+}(t,L)$ and is imposed by the passive laser cavity model.

**Figure 2:**
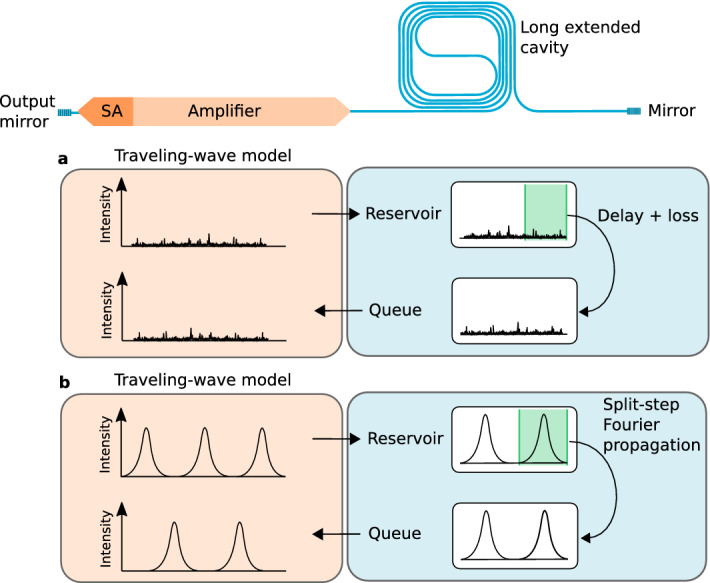
Mode-locked laser simulation flow, consisting of a traveling-wave model for the active region and a split-step Fourier propagation algorithm for the extended passive waveguide cavity. (**a**) In case no pulses are observed, e.g. at laser start-up, the split-step Fourier algorithm is not employed and the extended cavity is simply represented by a delay with some loss. (**b**) When pulses are observed, the split-step Fourier method is used and dispersive and nonlinear effects are accounted for.

### Passive waveguide model equations

The active laser sections are modeled with the aforementioned TWM, whereas the extended passive laser cavity is modeled either with a split-step Fourier method or a simple delay with loss, as is schematically illustrated in Fig. 2. The split-step Fourier method has been used extensively in literature and consists of a nonlinear- and dispersion operator that act alternately upon the propagating field^36,37,45^. Here we concisely describe the implementation used in our model, for further details we refer to the literature^35,37,46–48^. In this work, the linear losses, nonlinear losses due to two-photon and free-carrier absorption, second- and third-order dispersion, the Raman effect and third order nonlinear interactions stemming from the Kerr nonlinearity of the silicon waveguide are included through a generalized nonlinear Schrödinger equation of the form^35,37,46–48^

8 $$\begin{aligned} \frac{\partial E}{\partial z}-j\sum _{n=1}^{\infty }\frac{j^n \beta _{n}}{n!}\frac{\partial ^n E}{\partial t^n} =j(\gamma +\frac{1}{\omega _{0}}) \left[ E(z,t) \int _{0}^\infty R(t')|E(z,t-t')|^{2}dt' \right] -\frac{\alpha }{2}E-\frac{\sigma N_{c}}{2}E-jk_{c}k_{0}N_{c}E, \end{aligned}$$

where E(z,t) is the slowly varying pulse envelope (units of $\sqrt{W}$), z is the spatial variable and has the dimension of distance, $\beta _{n}$ represents the n-th order dispersion term and $\alpha$ denotes the linear losses. Furthermore, the nonlinear parameter is defined as $\gamma =\frac{n_{2}\omega _{0}}{A_{eff}c}+j\frac{\beta _{TPA}}{2A_{eff}}$, where $n_{2}$ is the material nonlinear coefficient, $A_{eff}$ is the effective mode area, c is the speed of light in vacuum, and $\beta _{TPA}$ is the two-photon absorption parameter. Furthermore, free-carrier absorption and dispersion are included through the free-carrier absorption coefficient $\sigma$, the free-carrier dispersion $k_{c}$, and the auxiliary equation that governs the time dependence of the free-carrier density $N_{c}$^47,48^

9 $$\begin{aligned} \frac{dN_{c}}{dt}=\frac{\beta _{TPA}}{2 \hbar \omega _{0} A_{eff}^{2}} |E|^4-\frac{N_{c}}{\tau _{FCA}}, \end{aligned}$$

where $\tau _{FCA}$ is the free-carrier lifetime in the waveguide and $\hbar \omega _{0}$ is the photon energy. Furthermore, the integral in Eq. (8) accounts for intrapulse Raman scattering through the nonlinear response function $R(t)=(1-f_{R})\delta (t)+f_{R}h_{R}(t)$ with $f_{R}$ the fractional contribution of the delayed Raman response to the nonlinear polarization and $h_{R}(t)$ the Raman response function, which can be approximated by an analytical function^35,49,50^

10 $$\begin{aligned} h_{R}(t)=\frac{\tau _{1}^{2}+\tau _{2}^{2}}{\tau _{1}\tau _{2}^{2}}e^{-t/\tau _{2}}sin(t/\tau _{1}) \end{aligned}$$

where $\tau _{2}=1/\Gamma _{R}$, $\tau _{1}=1/(\omega _{R}^{2}-\Gamma _{R}^{2})^{1/2}$, and $\Gamma _{R}/\pi \approx$105 GHz and $\omega _{R}/2\pi$=15.6 THz respectively determine the Raman-gain bandwidth and Raman shift in the silicon waveguide^50^.

Equation (8) can be solved using the well-known split-step Fourier method^35,37,46^. For the computation of the nonlinear term with the integral, a Runge-Kutta method can be employed, as elaborated in^35,49^. However, as pulses in semiconductor mode-locked lasers usually contain many optical cycles (i.e. pulse widths$>100\,\hbox {fs}$), it is acceptable to simplify Eq. (8) using a Taylor-series expansion, leading to^37^

11 $$\begin{aligned} \frac{\partial E}{\partial z}+\frac{j\beta _{2}}{2}\frac{\partial ^{2} E}{\partial T^{2}}-\frac{\beta _{3}}{6}\frac{\partial ^3 E}{\partial T^3}= j\gamma \left[ |E|^{2}E+\frac{j}{\omega _{0}} \frac{\partial }{\partial T}|E|^{2}E- T_{R}\frac{\partial |E|^{2}}{\partial T}\right] - \frac{\alpha }{2}E-\frac{\sigma N_{c}}{2}E-jk_{c}k_{0}N_{c}E, \end{aligned}$$

where $T_{R}\equiv \int _{0}^{\infty }tR(t)dt$ and a frame of reference moving with the pulse at the group velocity was introduced, i.e. T=t-z/$v_{g}$. This equation can be rewritten in the form $\frac{\partial E}{\partial z}=(\hat{D}+\hat{N})E$ where $\hat{D}$ is the operator that accounts for dispersion and losses and $\hat{N}$ accounts for nonlinearity^35,37^. The propagation over one step $\delta z$ can than be computed as

12 $$\begin{aligned} E(z+\delta z,T) \approx exp\left( \frac{\delta z}{2} \hat{D}\right) exp(\delta z \hat{N})exp\left( \frac{\delta z}{2} \hat{D}\right) E(z,T) \end{aligned}$$

where the $\hat{D}$ operator can be evaluated in the Fourier domain using a Fast-Fourier-Transform (FFT)^37^. A step size around ${100}\,\upmu \hbox {m}$ was employed for the split-step Fourier method, which is similar to earlier reported discretization steps^49^.

### Hybrid modeling strategy

To effectively propagate the output of the TWM in the passive waveguide model, a custom algorithm was developed. For the passive waveguide, a so-called *reservoir* and a *queue* data array are defined with a size equal to twice the propagation delay of the passive waveguide cavity divided by the TWM time stepsize, resembling respectively the forward and backward traveling waves in the passive cavity. In other words, the length of these arrays is in correspondance with the delay $\Delta T$ (in this case 359 ps) between light leaving the amplifier at the right side (see Fig. 2), and reaching the amplifier again at the right side after a roundtrip propagation through the silicon spiral waveguide cavity.

The forward propagating envelope of the TWM gradually fills the reservoir of the cavity, i.e. every iteration the last sample of the forward propagating envelope $A^{+}$ at the interface with the passive cavity (see Fig. 2) is sent to the reservoir. Simultaneously, a sample from the queue is concatenated to the backwards traveling envelope $A^{-}$ at the same interface. The forward propagating envelope $A^{+}$ hence continuously feeds the reservoir whereas the queue feeds the backward propagating envelope $A^{-}$. When the reservoir is full, the split-step Fourier method is activated to propagate (a number of) the reservoir samples. The split-step Fourier method is hence not called with every TWM iteration. The propagated samples are subsequently stored in the queue for continuation in the TWM.

To propagate the pulse, an appropriate time window has to be chosen to center the pulse, ensuring that energy is centralized in the window to comply with the periodic boundary conditions of the split-step Fourier method^37,51^. This avoids unphysical results and numerical instabilities^37^. A peak search algorithm is employed to detect any pulse-like patterns in the reservoir of the extended passive cavity. In case no peaks are detected, one can conclude that the laser either operates in continuous wave or is in a noisy (start-up) state. In these cases where no pulses are detected, the cavity is simply modeled with loss and a delay, as is shown in Fig. 2a. In case a single pulse is detected, a time slice is taken from the reservoir data and subsequently used as an input for the passive waveguide model. The slice is taken in such a way that the pulse is centered and can be readily propagated using the split-step Fourier method. Once the pulse is propagated, the resulting output is added to the queue. Finally, in case multiple pulses are detected in the reservoir, a time slice with one or more pulses is selected from the reservoir in a way that centralizes the signal energy in the slice as much as possible. This certifies that the intensity vanishes near the boundaries and warrants valid usage of the FFT in the split-step Fourier method. The resulting output is then again added to the queue that feeds the backward propagating wave of the TWM. As such, when pulsed behavior is observed, nonlinear and dispersive effects of the laser cavity are accounted for.

As expected, simulations confirm that after start-up the time slice used for split-step Fourier propagation is nearly equal to the entire reservoir size, i.e. the delay $\Delta T$=359 ps. Only during the start-up phase of mode-locking, a slightly smaller time slice can be observed (typically between 200 ps and 300 ps). Furthermore, the number of samples employed for the split-step Fourier propagation is equal to the number of samples selected from the reservoir as this avoids the need for interpolation. As such, the propagated signal samples in the queue can simply be concatenated to the backward propagating wave $A^{-}$ after applying a scaling factor to convert the field envelope in units of [$\sqrt{\mathrm{W}}$] to units of [$\sqrt{\mathrm{W}/\mathrm{m}^{2}}$].

It is observed that once steady-state mode-locking is reached, the split-step Fourier method is consistently employed for all pulses, as is conceptually shown in Fig. 2b. Moreover, simulations confirm that in case dispersion and nonlinearity of the passive waveguide are omitted, the hybrid model yields results identical to the TWM without the split-step Fourier approach. In case the cavity dispersion is anomalous, it can be worthwhile to consistently use the full reservoir for split-step Fourier propagation instead of a simple delay with loss. This allows one to account for the breakup of (quasi)-CW light or the amplification of noise through modulation instability. However, the current model is not optimized for continuous-wave-like operating points. Moreover, modeling pulsed regimes with nonvanishing backgrounds is currently outside the scope of the hybrid model owing to the periodic boundary conditions of the FFT.

**Figure 3:**
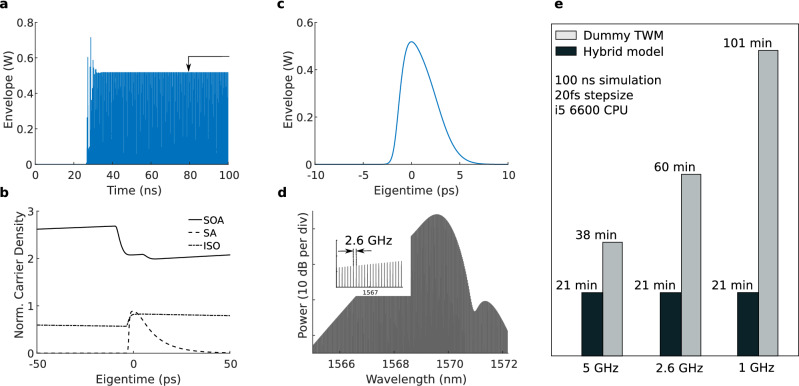
Mode-locked laser simulation example and benchmark. (**a**–**d**) Hybrid model output with a 20 fs stepsize and a 100 ns (260 roundtrips) duration. (**a**) Signal build-up at the output facet of the mode-locked laser. The arrow indicates the time instant of the corresponding normalized carrier densities (**b**) and pulse profile (**c**). (**d**) The optical comb spectrum corresponding with the generated pulse train. (**e**) Comparison of a dummy TWM and a hybrid model for a 5 GHz, 2.6 GHz and 1 GHz repetition rate. The hybrid model computation time is approximately invariant to the extended passive waveguide cavity size as it is not modeled by a slow TWM.

Figure 3a–d show the hybrid model results for the 2.6 GHz anti-colliding III–V-on-Silicon MLL. A simulation time of 100 ns (260 roundtrips) was used with a 20 fs stepsize. The model parameters are based on earlier work^48,52,53^ and are listed in Table 1. The real part of the nonlinear coefficient $\gamma _{NL}=\frac{n_{2}\omega }{A_{eff}c} \approx 69\,{\mathrm{m}}^{-1}{\mathrm{W}}^{-1}$, with $n_{2}$ the material nonlinear coefficient and $A_{eff}$ the effective mode area. The dispersion parameters $\beta _{2}=1.3$ ps$^{2}$/m and $\beta _{3}=0.0042$ ps$^{3}$/m were acquired using Lumerical^54^ based on the silicon waveguide dimensions specified in^38^.

An injection current of 45 mA was found to correspond with fundamental mode-locking. At this operating point, the pulse train converges after approximately 40 ns (100 roundtrips), as can be seen in Fig. 3a. The black arrow in Fig. 3a indicates the time instant used to acquire the carrier density profiles, shown in Fig. 3b, and the individual pulse profile, shown in Fig. 3c. The output pulse has an energy of 2.11 pJ and a full-width at half maximum (FWHM) of 3.68 ps. These values are in in line with experimental results where pulses with energies on the order of 1 pJ are observed and autocorrelation measurements indicate pulsewidths around 3 ps^38,55^. The carrier densities were normalized with respect to the transparency carrier density and were monitored in the middle of the amplifier, saturable absorber and isolation sections. The carrier density in the saturable absorber quickly saturates with the incoming pulse but recovers fast compared to the SOA. Furthermore, as the SOA is close to the output facet, the pulse propagates twice through the SOA with a short delay in between, resulting in two subsequent dips in the SOA carrier density, as can be seen in Fig. 3b. The comb spectrum, shown in Fig. 3d, exhibits a dip around 1571 nm caused by the cavity third-order nonlinearity. In addition, part of the comb spectrum is slightly red-shifted resulting from the Raman effect. The line spacing between the comb teeth is 2.6 GHz, corresponding with the pulse repetition rate of the laser.

**Table 1:** Parameters used. SOA semiconductor optical amplifier, SA saturable absorber, ISO isolation region in between the SOA and SA.

Meaning	Symbol	Value	Units
Wavelength	$\lambda$	1.57	$\upmu$m
Group index	$n_{g}$	3.85	–
Effective index	$n_{eff}$	3	–
Transparency carrier density	$N_{t}$	$8.7\times 10^{17}$	cm$^{-3}$
MQW confinement factor	$\Gamma$	0.075	–
MQW mode area	$A_{MQW}$	$0.54 \times 10^{-12}$	m$^{2}$
Carrier lifetime	$\tau _{SOA}$; $\tau _{SA}$; $\tau _{ISO}$	1; 10$^{-2}$; 1	ns
Gain constant	$\chi _{0,SOA}$; $\chi _{0,SA}$; $\chi _{0,ISO}$	0.07; 0.48; 0.07	–
Intraband relaxation rate	$\gamma _{SOA}$; $\gamma _{SA}$; $\gamma _{ISO}$	$4 \times 10^{12}$; $8 \times 10^{12}$; $8 \times 10^{12}$	s$^{-1}$
Filter bandwidth	$\Delta f$	2	THz
Section length	$L_{SOA}$; $L_{SA}$; $L_{ISO}$	850; 60; 30	$\upmu$m
Top band frequency	$\Omega _{T}$	$90 \times 10^{12}$	rad s$^{-1}$
SA Bandgap offset	$\Omega _{g}$	5	THz
Active region internal losses rate	$\beta$	$2.56 \times 10^{11}$	s$^{-1}$
Injection current	*I*	45	mA
Silicon waveguide mode area	$A_{Si}$	$0.29 \times 10^{-12}$	m$^{2}$
Second-order dispersion	$\beta _{2}$	1.3	ps$^{2}$/m
Third-order dispersion	$\beta _{3}$	0.0042	ps$^{3}$/m
Silicon Kerr nonlinearity	$n_{2}$	$5\times 10^{-18}$	m$^{2}$W$^{-1}$
Silicon waveguide losses	$\alpha$	0.7	dB/cm
Two-photon absorption silicon	$\beta _{TPA}$	0.6	cm/GW
Free-carrier dispersion silicon	$k_{c}$	$1.35\times 10^{-27}$	m$^{3}$
Free-carrier lifetime silicon	$\tau _{FCA}$	1	ns
Free-carrier absorption silicon	$\sigma$	$1.45\times 10^{-21}$	m$^{2}$
Facet reflectivity	$r_{1}$; $r_{2}$	0.5; 0.99	–

Figure 3e shows a comparison of the hybrid model and a dummy TWM without split-step Fourier propagation of identical complexity for different MLL repetition rates. In the dummy TWM case, the field propagation through the passive waveguide cavity is calculated in a traveling-wave fashion to emulate the incorporation of dispersive and nonlinear effects. However, actual dispersive and nonlinear effects are omitted in the dummy TWM as it solely serves as a reference for the simulation time. Note that if one does not desire to include dispersion and nonlinearity in practice, one can use a traveling-wave model with a simple boundary condition for the passive waveguide cavity, leading to a simulation time comparable to that of the hybrid model. The MLL was simulated for 100 ns with a timestep of 20 fs on a standard desktop with an i5-6600 Central Processing Unit (CPU). The MATLAB script of the hybrid model takes around 20 minutes to complete, almost independently of the passive cavity size. Note that the considered hybrid model is solely prototyped in MATLAB and does not rely on any custom memory allocation or parallelization. Switching to a low-level programming language could therefore provide for a massive speed-up, as was also exploited by earlier work^19,20^. As the TWM dominates the total simulation time of the hybrid model, the split-step Fourier algorithm for the passive cavity only marginally increases the computation time with decreasing repetition rate. The classical TWM on the other hand, in which also the passive laser cavity is modeled with a traveling-wave method, requires significantly more time, as is indicated in Fig. 3e. Moreover, the simulation time of the classical TWM rapidly increases with decreasing repetition rate because the TWM complexity directly scales with the laser cavity size. This drawback highlights the benefits of a hybrid modeling strategy: it allows to include complex dispersive and nonlinear effects of the extended passive laser cavity with minimal computational penalty. This is particularly valuable for integrated MLLs with a low repetition rate^4,10,12^, as the impact of nonlinear and dispersive effects of the cavity becomes apparent in these devices and the simulation time with existing TWMs can become impractically long.

**Figure 4:**
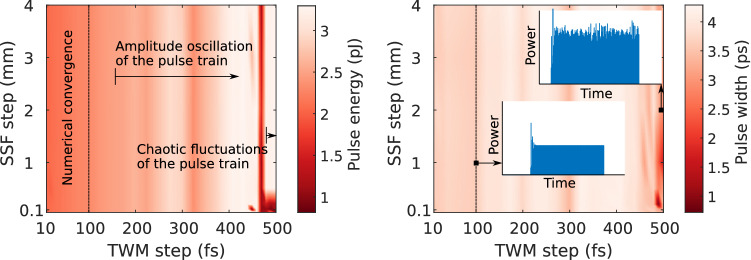
Stability analysis of the hybrid mode-locked laser model for an injection current of 45 mA. Map of the pulse energy (left) and of the pulsewidth (right) as a function of the discretization step of the TWM and the stepsize of the split-step Fourier propagation.

Figure 4 depicts a map of the output pulse energy (left) and the output pulse width (right) as a function of the traveling-wave discretization step and the split-step Fourier step. One finds that the stability of the TWM model degrades gradually without an abrupt transition to a numerically unstable regime. For a TWM step below approximately 100 fs, the model yields no discernible differences and convergence is achieved. However, when the TWM stepsize is further increased, the amplitude of the pulse train acquires an oscillatory fluctuation. Furthermore, for very large stepsizes, approaching 500 fs, the amplitude fluctuations appear to be chaotic and the pulse train becomes unstable, as can be seen from the inset on the right of Fig. 4. Moreover, due to the coarse TWM grid, the pulse envelope is poorly sampled, leading to strongly distorted pulse shapes with sharp edges. Finally, it is observed that the model is robust with regard to the split-step Fourier (SSF) step size. Although the pulse energy and pulse width remain nearly identical for increasing SSF steps, the comb spectrum starts to deviate when the step size exceeds several millimeters. For all simulations in the manuscript, a step of approximately ${100}\,\upmu \hbox {m}$ was employed for the split-step Fourier method.

### Impact of dispersion, third-order nonlinearity and nonlinear losses

It is well known that the complex interplay of dispersion and nonlinearity can greatly affect the pulse generation of MLLs. Even more so, careful management of these properties has lead to various types of soliton lasers, where the balance between quadratic^56,57^ or higher-order dispersion^58^ with nonlinearity have allowed for stable ultrashort pulse generation. Although solitons have traditionally been produced with fiber MLLs, they can also arise in chip-scale devices^58^. Furthermore, even for existing integrated MLLs which do not target solitary operation, dispersive and nonlinear effects of the passive waveguides can significantly affect the properties of the pulse train, in particular for long waveguide cavities.

**Figure 5:**
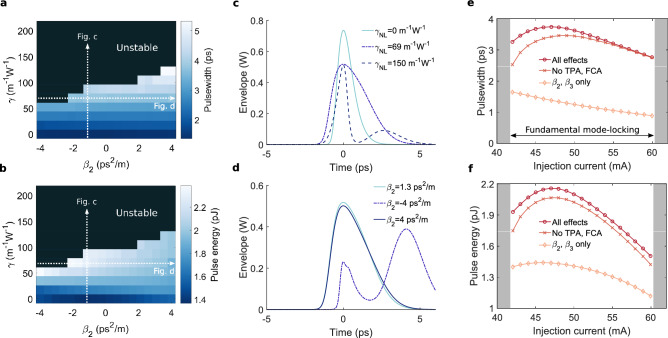
Impact of nonlinearity and dispersion of the extended passive laser cavity on the MLL performance and stability. Map of the pulse width (**a**) and pulse energy (**b**) as a function of GVD and Kerr nonlinearity. Stable fundamental mode-locking is predominantly observed for sufficiently small nonlinearities and normal dispersion. Output pulse for different nonlinearities and $\beta _{2}=1.3\,{\mathrm{ps}}^{2}/{\mathrm{m}}$ (**c**), and for different cavity dispersions with $\gamma _{NL}= 69\,{\mathrm{m}}^{-1}{\mathrm{W}}^{-1}$ (**d**). The corresponding operating regions on the maps (**a**,**b**) are indicated. Output pulse width (**e**) and pulse energy (**f**) as a function of injection current for $\gamma _{NL}= 69\,{\mathrm{m}}^{-1}{\mathrm{W}}^{-1}$ and $\beta _{2}=1.3\,{\mathrm{ps}}^{2}/{\mathrm{m}}$ (**e**).

A map of the pulse energy and the FWHM pulse duration as a function of Kerr nonlinearity and group-velocity dispersion of the passive cavity is shown in Fig. 5a and b respectively. The injection current of the III–V-on-Silicon MLL model was fixed at 45 mA. Stable fundamental mode-locking can primarily be observed at low nonlinearities, while fundamental mode-locking ceases for anomalous dispersive and/or highly nonlinear operating points.

The output pulses under normal dispersion $\beta _{2}=1.3\,{\mathrm{ps}}^{2}/{\mathrm{m}}$ and Kerr nonlinearities $\gamma _{NL}= 0\,{\mathrm{m}}^{-1}{\mathrm{W}}^{-1}$, $\gamma _{NL}= 69\,{\mathrm{m}}^{-1}{\mathrm{W}}^{-1}$ and $\gamma _{NL}= 150\,{\mathrm{m}}^{-1}{\mathrm{W}}^{-1}$ are depicted in Fig. 5c. By introducing a positive nonlinear coefficient $n_{2}$, the Self-Phase Modulation (SPM)-induced chirp leads to a spectral red-shift for the leading edge of the pulse and a blue-shift for the trailing edge. Under normal dispersion, this leads to enhanced pulse broadening, as can be observed in Fig. 5c and in the map Fig. 5a. Furthermore, the SPM-induced chirp in combination with normal dispersion in the silicon waveguide shapes the pulse as such that it becomes rectangularly shaped with sharper leading and trailing edges, a phenomenon known as optical wave breaking^59^. This cavity pulse shaping is however not apparent from the output pulse plot as the pulse shape is also strongly affected by the amplifier and absorber sections, smoothing the sharpened pulse edges. In contrast, under anomalous dispersion the pulse duration reduces with increasing SPM. For sufficiently strong SPM, the pulseshape is distorted and experiences a temporal oscillation around the pulse peak, eventually leading to an unstable pulse train as is the case for $\gamma _{NL}= 150\,{\mathrm{m}}^{-1}{\mathrm{W}}^{-1}$ in Fig. 5c. The output pulses for different Group-Velocity Dipsersion (GVD) values and for a nonlinearity $\gamma _{NL}= 69\,{\mathrm{m}}^{-1}{\mathrm{W}}^{-1}$ are shown in Fig. 5d. The cases $\beta _{2}=1.3\,{\mathrm{ps}}^{2}/{\mathrm{m}}$ and $\beta _{2}=4\,{\mathrm{ps}}^{2}/{\mathrm{m}}$ lead to stable mode-locking and yield a nearly identical pulse train. On the other hand, changing the $\beta _{2}=-4\,{\mathrm{ps}}^{2}/{\mathrm{m}}$ results in a chaotic pulse train with varying pulse amplitudes and the emergence of satellite peaks.

Figure 5e and f depict the pulse width and pulse energy for the case (1) where all aforementioned cavity effects are considered, in case (2) two-photon- and free-carrier absorption are neglected and for case (3) only dispersion is considered without nonlinearity, Raman effect or nonlinear absorption. Stable fundamental mode-locking is achieved for injection currents between 42 and 60 mA. For lower injection currents, the MLL operates in a noisy or Q-switched state, whereas at high injection currents, satellite peaks arise in the trailing edge of the pulse, leading to chaotic or seemingly harmonicly mode-locked operation. Omitting the nonlinearity leads to a slight enhanced stability range, ranging from 42 to 62 mA. For the considered operating point, the Kerr nonlinearity hence slightly deteriorates the stability of the MLL and advances the transition to an unstable pulse train regime. As a simulation time of 100 ns was used, it is feasible that some stable fundamental operating points lay outside the depicted region but require long convergence times. Furthermore a number of stable attractors are potentially not accessible by self-starting the mode-locked laser and may require some form of excitation such as a pulse injection.

For case (1) and (2) the pulsewidth increases up to an injection current around 47 mA, after which the pulse width monotonically decreases. As the pulse peak power is observed to be approximately invariant with the injection current, the pulse energy follows a similar trend as the pulsewidth, reaching the maximal pulse energy at an injection current around 47 mA. As can be expected, the incorporation of two-photon- and free-carrier absorption leads to a reduced output power, on average resulting in a 5% lower pulse energy. As for the example considered here the effective mode area $A_{eff}$ is relatively large and the pulse energies are low (< 2.2 pJ), it is presumed that nonlinear losses play a salient role in chip-scale mode-locked lasers with a silicon waveguide cavity. Switching to a silicon-nitride platform could hence be a valuable alternative to eliminate the detrimental effects of two-photon- and free-carrier absorption altogether^60^. In case (3), the pulse width and peak power respectively decrease and increase with injection current. Moreover, although the pulse is significantly shorter compared to cases (1) and (2), the pulse energy is greatly reduced as well, leading to a lower average output power compared to the cases where the third-order nonlinearity is included.

**Figure 6:**
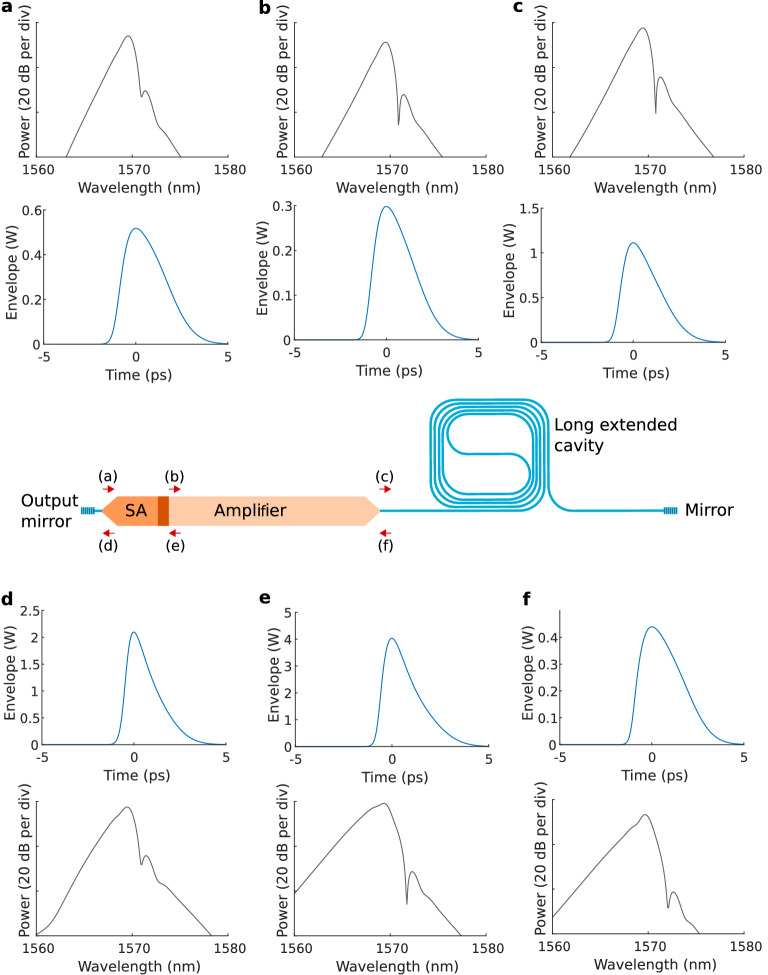
Temporal and spectral pulse evolution in the laser cavity. Pulse before the SA, after the spectral filter (**a**); after propagating through the SA and isolation section (**b**); before split-step Fourier propagation (**c**); after the SA, before the spectral filter and mirror (**d**); after amplification (**e**) and after propagation through the extended silicon cavity (**f**).

The temporal and spectral evolution of the pulse in the laser cavity is depicted in Fig. 6. The pulse and the corresponding spectrum are shown at 6 locations along the laser cavity to visualize the impact of the SA, SOA and extended silicon waveguide cavity. The spectrum after split-step Fourier propagation is strongly broadened and red-shifted and reveals a subtle oscillatory structure at the peak, as can be expected based on the SPM-induced nonlinear phase shift^61^. Furthermore, the spectral filter turns out to significantly affect the pulse spectrum and hence plays an essential role for stabilization.

## Conclusions

We have demonstrated a hybrid modeling strategy for semiconductor-based MLLs that combines the traveling-wave modeling technique for the semiconductor laser sections with a split-step Fourier method for the extended passive waveguide cavity. This novel approach paves to way to include a wide range of physical phenomena, such as the semiconductors physics of the SOA and SA as well as the dispersive and nonlinear properties of the extended passive laser cavity while simultaneously minimizing the model’s computational workload. The impact of dispersion, third-order nonlinearity and nonlinear losses on the pulse train and stability of a 2.6 GHz III–V-on-Silicon MLL was shown, hereby highlighting the importance to include these effects. We believe such a hybrid modeling strategy is particularly valuable to study low-repetition-rate MLLs, as for these devices dispersive and nonlinear effects of the long extended cavity can become dominant.

Compared to mode-locked laser models based on delay differential equations (DDEs)^25–27^, the traveling-wave approach does not presume a ring-cavity geometry with unidirectional propagation. Unidirectional ring-cavity mode-locked lasers do not exist in practice and are hence merely an idealization. Furthermore, in some cases it might be desirable to account for reflections at various interfaces in the semiconductor sections of the mode-locked laser. Moreover, DDEs do not incorporate spatial effects such as spatial hole burning, whereas TWMs naturally include such phenomena^19,20^. In addition, the split-step Fourier method easily incorporates other potentially relevant effects of the extended passive waveguide such as the Raman effect. Mode-locked laser models based on DDEs have yet to demonstrate this ability. While DDE-based models can offer a powerful alternative, they hence exhibit a slightly reduced physical accuracy^62,63^. We believe a hybrid model therefore offers a valuable complementary modeling technique to DDE-based approaches.

Furthermore, given the low computational complexity, the presented approach allows to conduct extensive parametric studies and the exploration of novel operating regimes such as chip-scale soliton mode-locking. Such studies could advance the understanding of the mode-locking dynamics and lead to a new generation of improved chip-scale MLL devices.

## Data Availability

All MATLAB code of the hybrid mode-locked laser model can be found on GitHub: https://github.com/stijncuyvers/HybridMLLmodel-ScientificReportsPaper.
